# Supplementary figures and images for: Biological age threshold is associated with symptomatic knee osteoarthritis risk in chinese adults: Insights from machine learning analysis of a national cohort
Source: PLoS One. 2025 Dec 17;20(12):e0335250. doi: 10.1371/journal.pone.0335250 (PMC12711007; doi:10.1371/journal.pone.0335250)

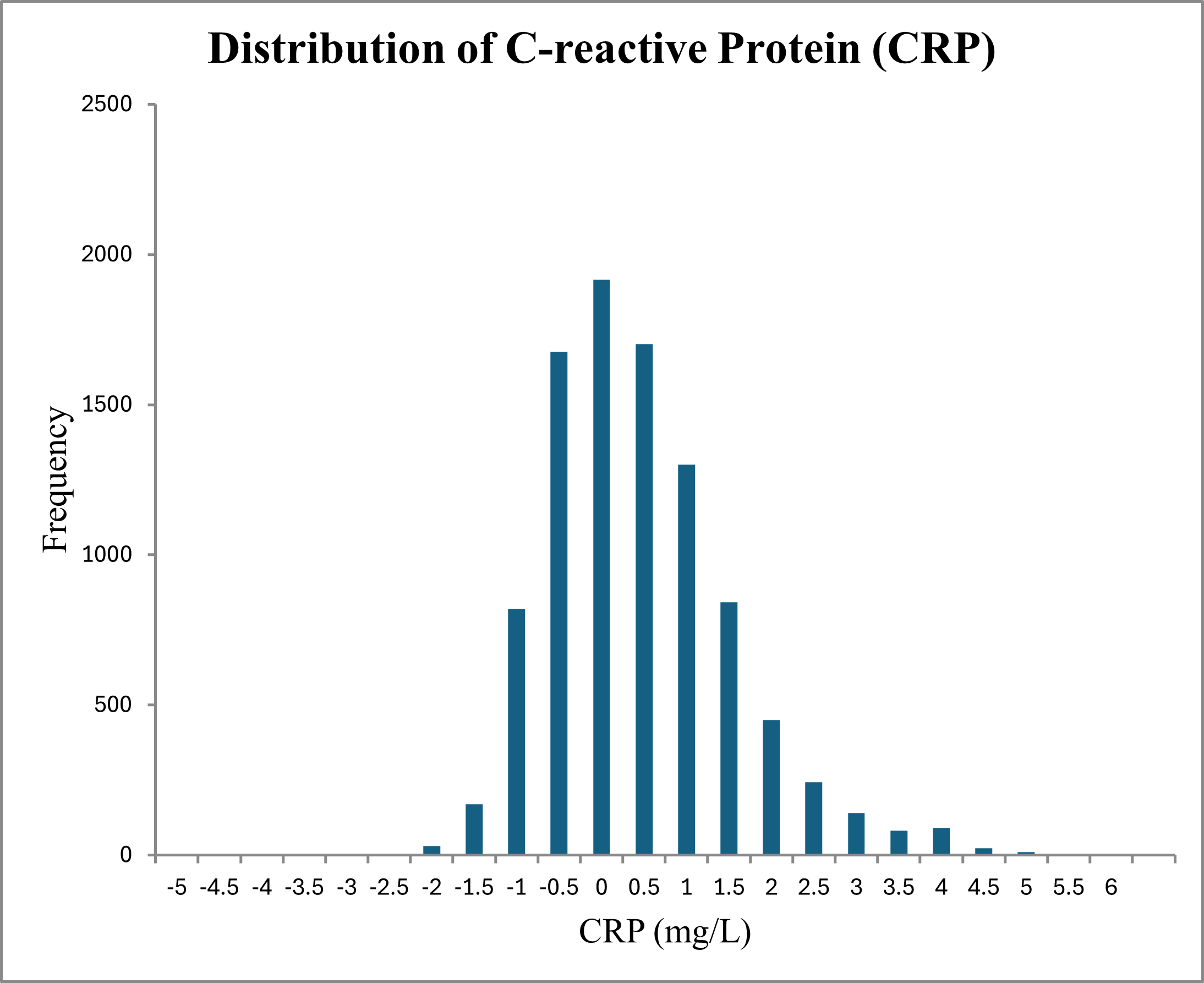

Supplement: S1 Fig — (TIF) [file pone.0335250.s001.tif]

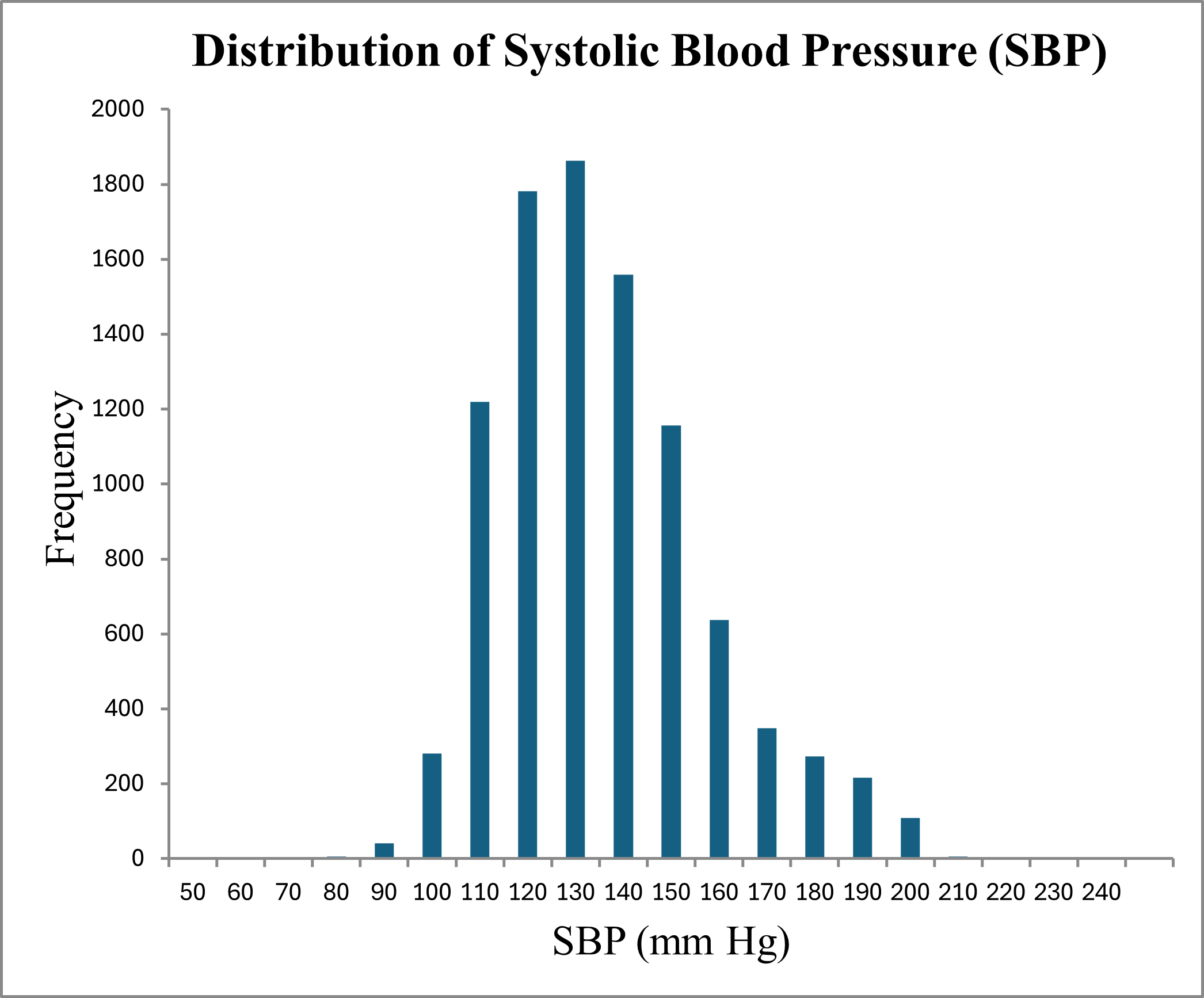

Supplement: S2 Fig — (TIF) [file pone.0335250.s002.tif]

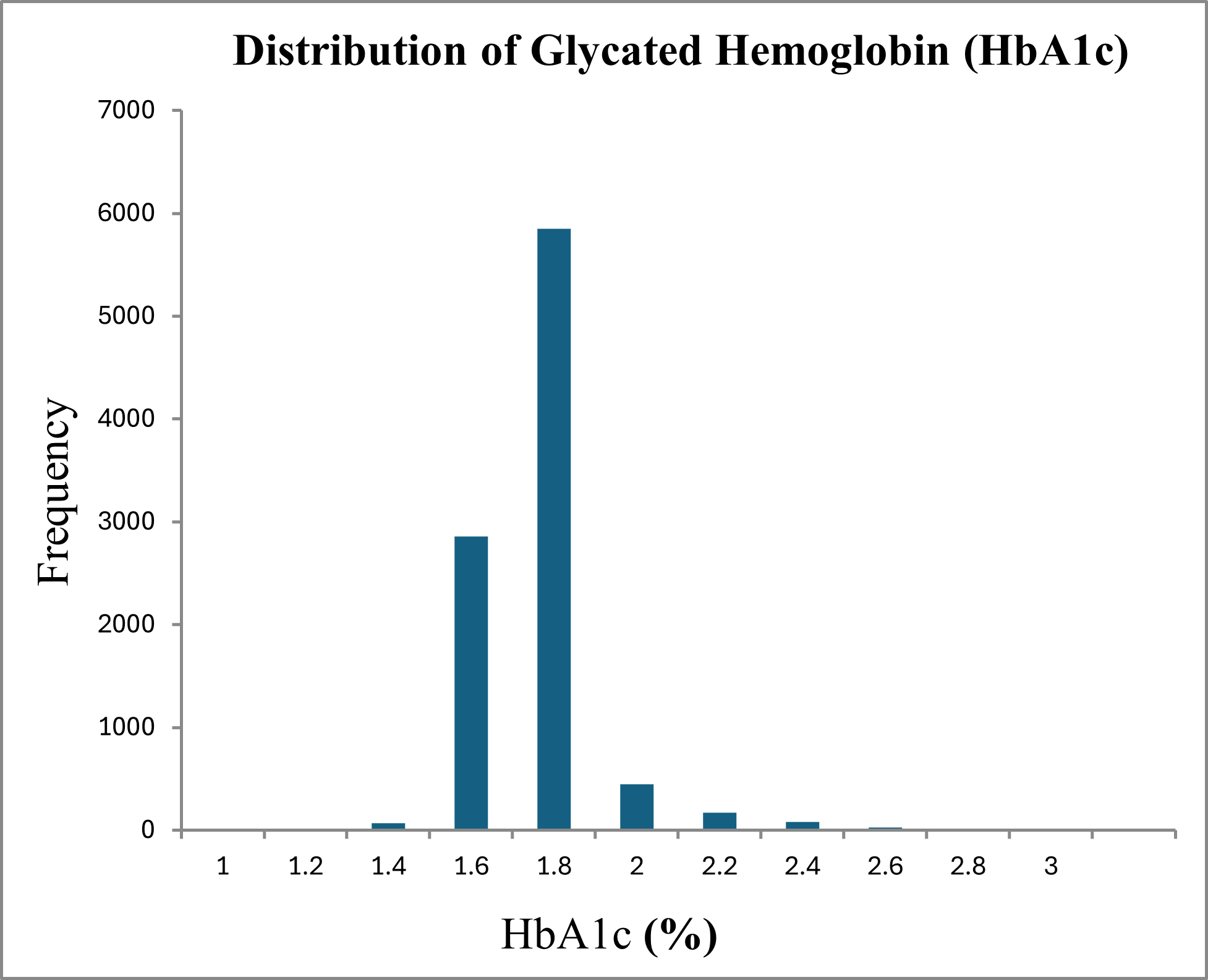

Supplement: S3 Fig — (TIF) [file pone.0335250.s003.tif]

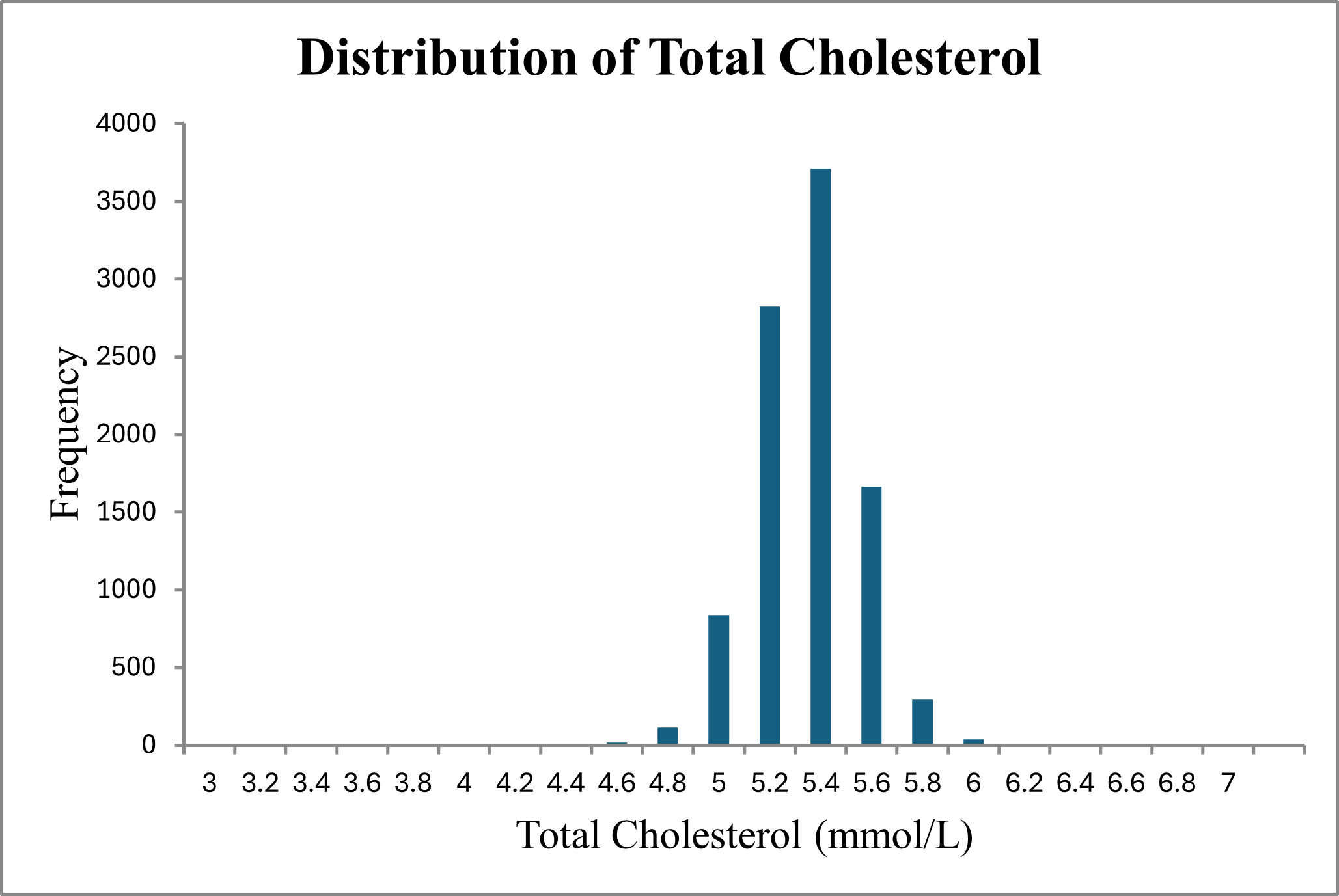

Supplement: S4 Fig — (TIF) [file pone.0335250.s004.tif]

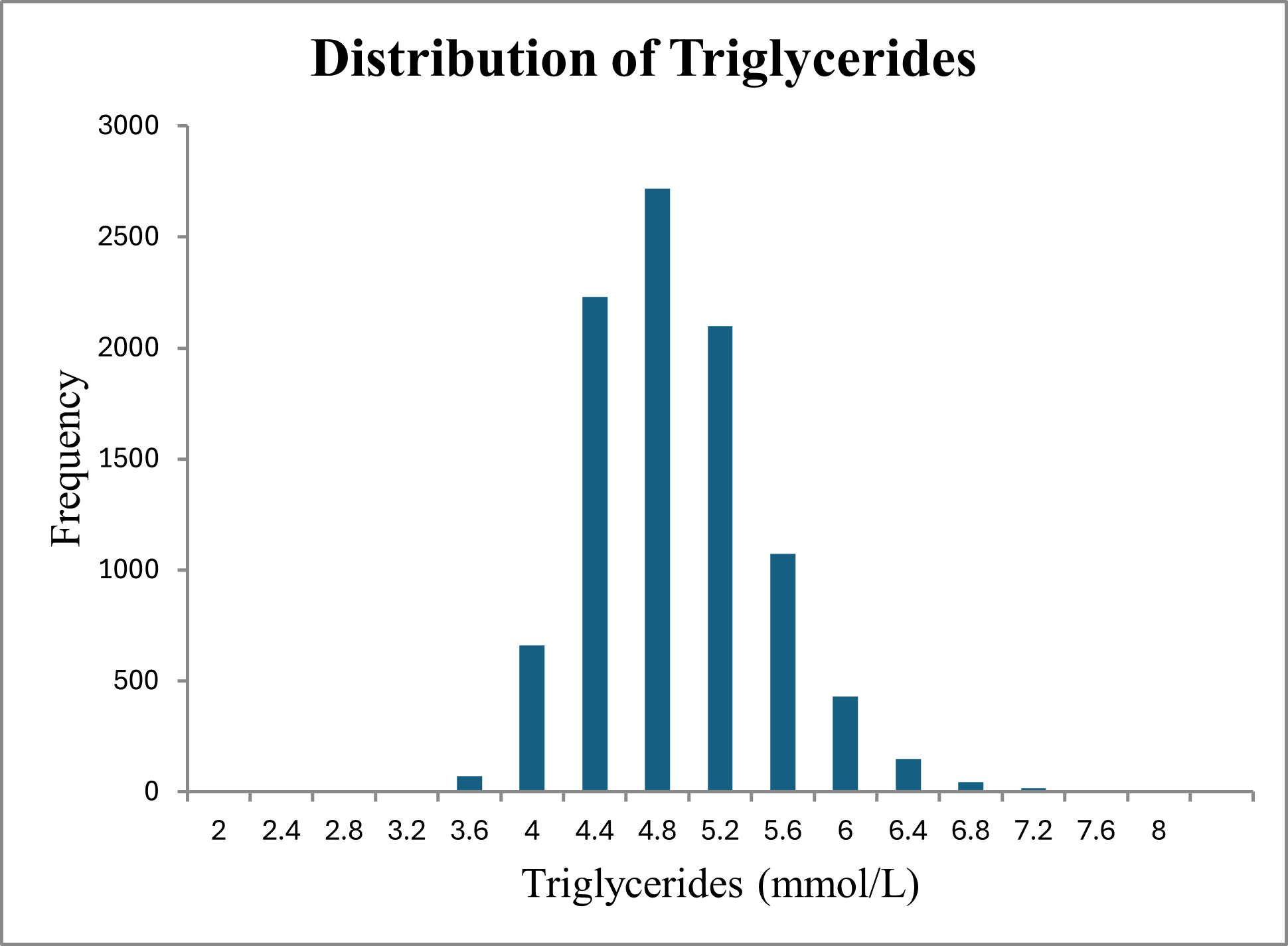

Supplement: S5 Fig — (TIF) [file pone.0335250.s005.tif]

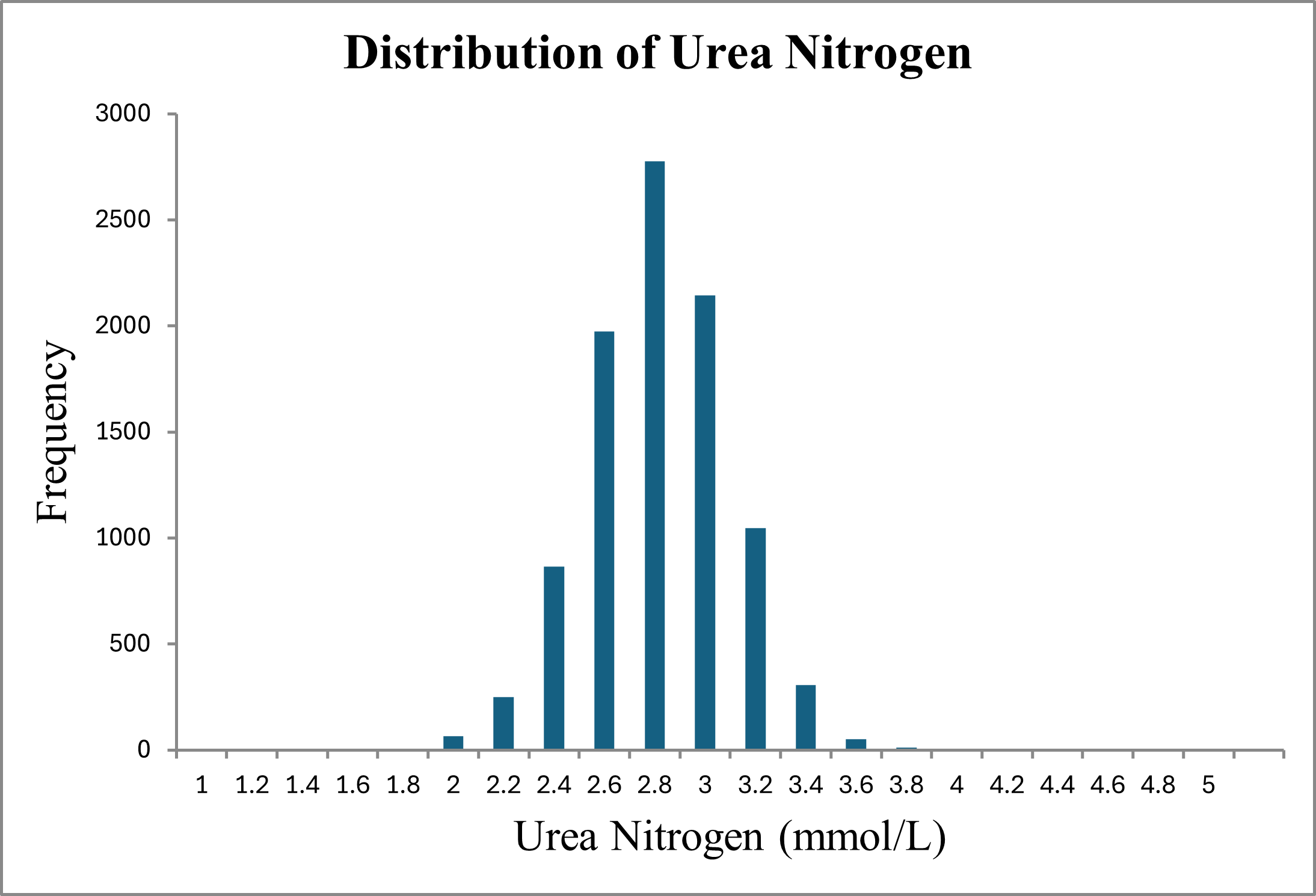

Supplement: S6 Fig — (TIF) [file pone.0335250.s006.tif]

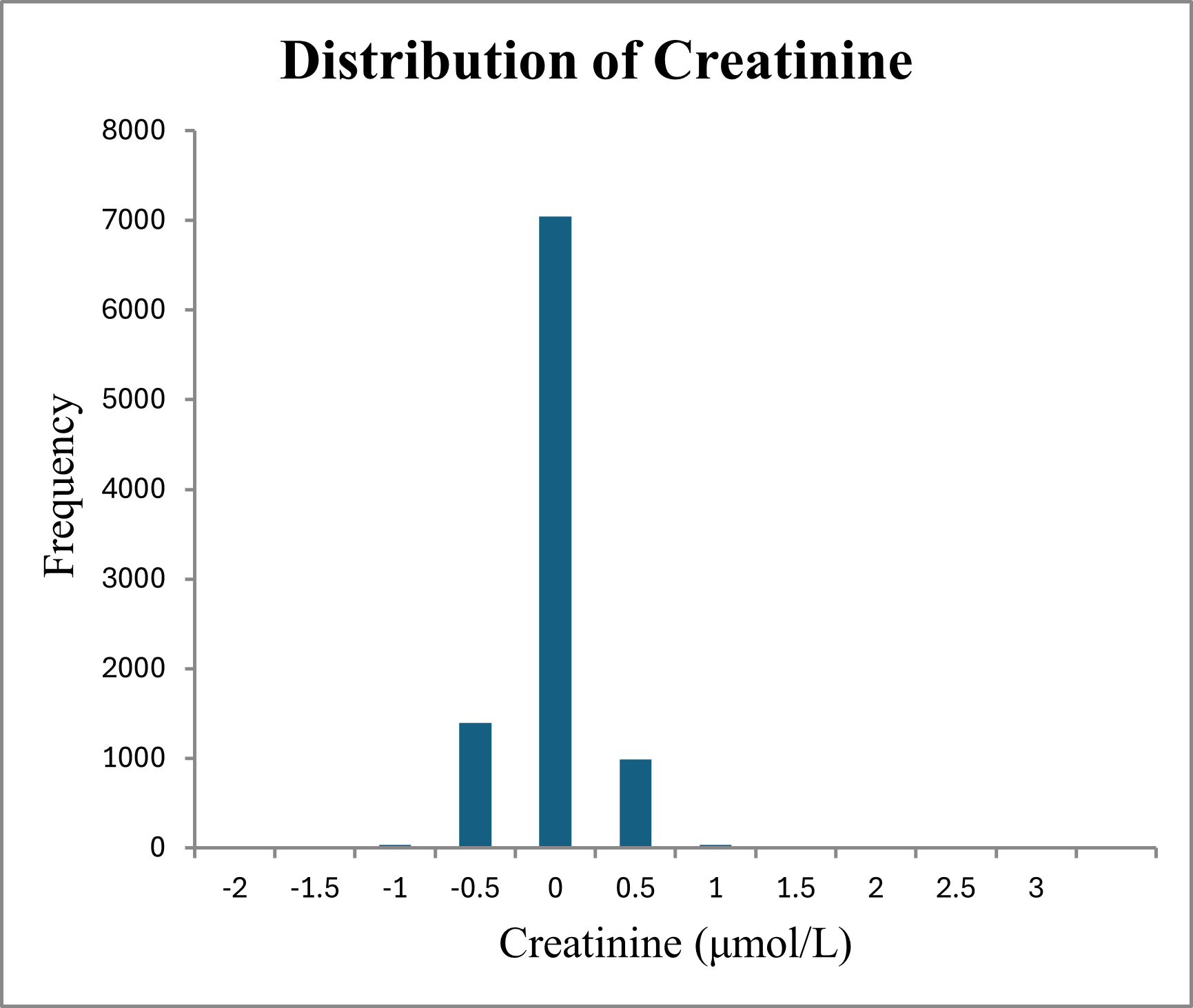

Supplement: S7 Fig — (TIF) [file pone.0335250.s007.tif]

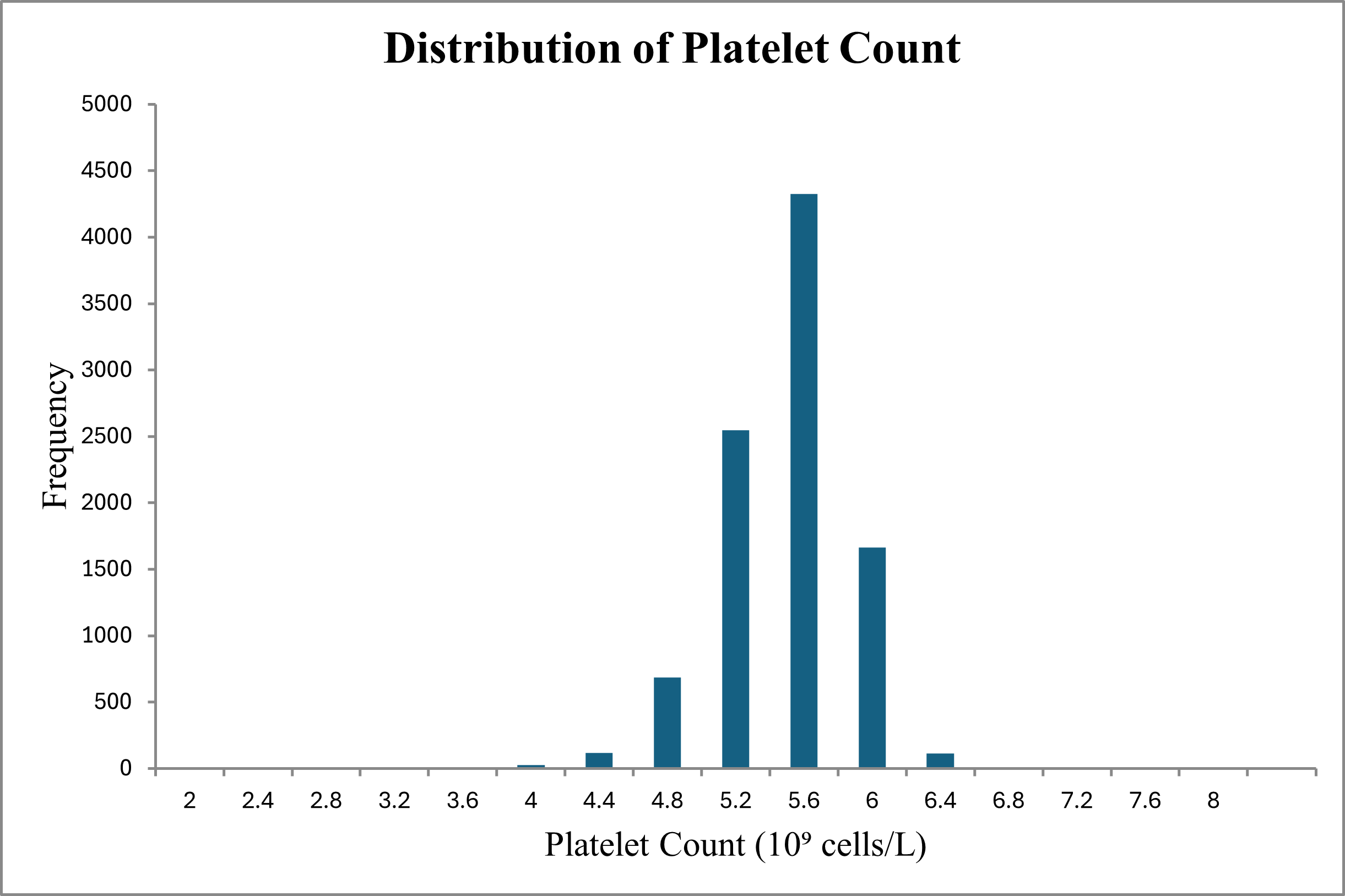

Supplement: S8 Fig — (TIF) [file pone.0335250.s008.tif]

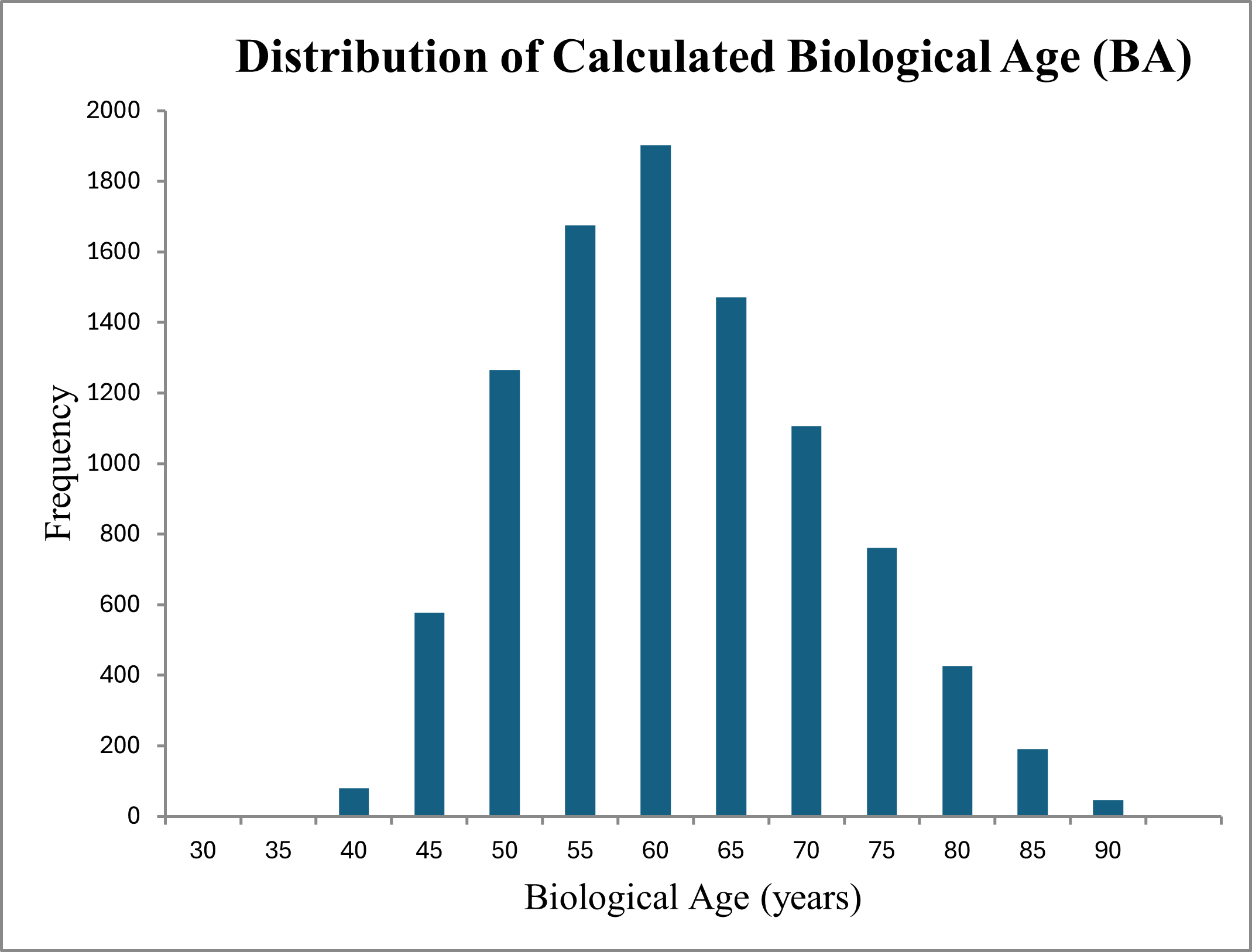

Supplement: S9 Fig — (TIF) [file pone.0335250.s009.tif]
