# Supplementary material for: Biological age threshold is associated with symptomatic knee osteoarthritis risk in chinese adults: Insights from machine learning analysis of a national cohort
Source: PLoS One. 2025 Dec 17;20(12):e0335250. doi: 10.1371/journal.pone.0335250 (PMC12711007; doi:10.1371/journal.pone.0335250)
Supplement: Attachment — Submitted filename: Review comments.docx [file pone.0335250.s012.docx]

Summary:

The authors investigated the association between biological age (BA) and symptomatic knee osteoarthritis (KOA) using data from the CHARLS cohort. They applied logistic regression, restricted cubic splines, and machine learning models (XGBoost, LightGBM, etc.) to explore the predictive value of BA. The study reports a non-linear association, with KOA risk accelerating after approximately 66.7 years of BA. XGBoost achieved the best predictive performance, and SHAP confirmed BA as the most influential factor. The authors conclude that BA is a promising biomarker for risk stratification of KOA.

1. Study Design:

The cross-sectional design does not allow causal inference, yet parts of the conclusion suggest predictive or causal implications.

Recommendation: Reframe claims to emphasize “association” rather than “prediction/causality.”

Sample Selection and Representativeness:

From ~38,800 participants, only 9,505 remained, and finally 1,000 KOA cases were analyzed. The selection process may introduce bias.

Recommendation: Provide detailed explanation on exclusion criteria, missing data handling, and sensitivity analysis.

2. KOA Diagnosis Criteria:

Symptomatic KOA was defined solely by self-reported physician diagnosis with pain, lacking imaging confirmation (e.g., KL grading).

Recommendation: Discuss the risk of misclassification in greater detail and, if possible, validate findings against other diagnostic definitions.

3. Biological Age Calculation

Only 8 biomarkers were used instead of the standard 12 in KDM-BA. Although justified with prior validation, potential bias is not sufficiently addressed.

Recommendation: Provide comparative or sensitivity analysis, and acknowledge limitations more explicitly.

4. Machine Learning Methodology

The sample size (1,000 KOA cases) may be limited for complex models, raising concerns of overfitting. No external validation dataset was used.

Recommendation: Add stronger justification for model selection, expand on overfitting prevention, and clarify whether performance was robust across subsets.

5. Discussion and Interpretation

The discussion overstates the clinical applicability of BA without sufficient consideration of cost, feasibility, and generalizability.

Recommendation: Temper the claims and include comparison with international studies.

6. Minor Concerns

Some terms in the abstract and conclusion (e.g., “predicts,” “causal”) should be replaced with more cautious wording.

Clarify covariates included in each logistic regression model table to improve transparency.

Figures (e.g., RCS, SHAP plots) should include clearer axis labels and clinical interpretation.

Data availability statement should specify exact CHARLS dataset versions used.

The manuscript addresses an important and novel question with innovative use of BA and machine learning. However, substantial revisions are required to address methodological transparency, diagnostic accuracy, and interpretation of findings. I recommend major revision before reconsideration.
