# Supplementary material for: Biological age threshold is associated with symptomatic knee osteoarthritis risk in chinese adults: Insights from machine learning analysis of a national cohort
Source: PLoS One. 2025 Dec 17;20(12):e0335250. doi: 10.1371/journal.pone.0335250 (PMC12711007; doi:10.1371/journal.pone.0335250)
Supplement: Attachment — Submitted filename: Response to Reviewers.doc [file pone.0335250.s013.doc]

**Response to Reviewers**

Dear Editors and Reviewers:

We would like to thank the editors and reviewers’ work devoted to our manuscript entitled " Biological Age Threshold is Associated with Symptomatic Knee Osteoarthritis Risk in Chinese Adults: Insights from Machine Learning Analysis of a National Cohort" (Sub ID: PONE-D-25-41030). The comments are valuable and very helpful for revising and improving our paper. We have studied the comments carefully and made corrections that we hope to meet with approval. The changes we have made are highlighted with blue background in the marked revised manuscript.

Each concern is discussed in detail below. We have made slight revisions to the title, and these revisions were performed to address the reviewers’ concerns. Thank you again for allowing us to resubmit our manuscript for your consideration.

**Detailed Responses to the Editor and Reviewer**

**Response to Editor:**

**Comment 1:**

*The introduction, while informative, is somewhat lengthy. Key ideas could be presented more concisely by merging overlapping sentences (e.g., lines 59–73 and 86–91, which both emphasize the burden and necessity theme).*

**Response**: Thank you for pointing out the redundancy in the introduction, particularly the overlapping emphasis on disease burden and the necessity of improved risk stratification. To address this, we have merged the content in Lines 59–73 (which outlines the global burden of KOA) and Lines 86–91 (which reiterates the need for better risk assessment). Specifically, we combined key data—including “KOA ranking as the 11th leading cause of disability globally, affecting approximately 3.8% of the world’s population” and “an estimated 37.35 million Chinese adults aged ≥60 years living with symptomatic KOA”—into a single concise statement. We then removed repetitive assertions about “substantial disease burden” and “urgent need for risk stratification”, while preserving the core logic that links KOA’s public health impact to the demand for enhanced assessment tools. This revision has streamlined the narrative and eliminated unnecessary verbosity.

**Comment 2:**

*Certain sections read as a list of facts rather than a connected narrative. For example, after presenting KOA prevalence and burden, it would be more effective to transition directly into why BA matters compared to CA, instead of repeating burden-related points.*

**Response**: Thank you for highlighting the need to strengthen narrative flow and avoid disjointed factual enumeration. We have restructured the transition after presenting KOA prevalence and burden: instead of repeating points about disease burden, we now move directly from the epidemiological data (e.g., prevalence in China and globally) to the limitations of CA as a risk factor—specifically, its inability to capture interindividual aging heterogeneity. This leads naturally to the introduction of BA as a more precise measure that integrates systemic biomarkers, creating a logical sequence: “epidemiological context → limitation of traditional markers → value of novel markers”. This revision eliminates the “list-like” feel and ensures a cohesive progression of ideas.

**Comment 3:**

*The mechanistic explanation of SASP (lines 82–86) is too detailed for the introduction. It could be shortened to a high-level overview with supporting citations, while the in-depth mechanistic pathways are better suited for the Discussion section.*

**Response**: Thank you for guiding us on the appropriate division of content between the introduction and discussion sections. We have simplified the mechanistic description of the senescence-associated secretory phenotype (SASP) in Lines 82–86 of the introduction: instead of detailing molecular pathways (e.g., chondrocyte matrix synthesis decline, upregulation of IL-6 and TNF-α), we now provide a high-level overview: “Biologically, BA may contribute to KOA pathogenesis through cellular senescence and the senescence-associated secretory phenotype (SASP)” (with the original citation [12] retained). The detailed mechanistic content—including the role of SASP in disrupting cartilage metabolism and promoting inflammation—has been relocated to the “Mechanistic Links Between BA and KOA” subsection of the Discussion, where it is integrated with other evidence (e.g., oxidative stress, chronic inflammation) to form a comprehensive analysis. This adjustment ensures the introduction focuses on framing the research gap rather than delving into specialized mechanistic details.

**Comment4:**

*The phrase “mitigating the burden of KOA” appears multiple times; rephrasing or reducing its repetition would improve clarity and flow.*

**Response**: Thank you for drawing attention to this repetitive phrasing, which we have revised to improve readability. We systematically reviewed the manuscript and replaced “mitigating the burden of KOA” with contextually appropriate alternatives:

1. In the Introduction, it is rephrased as “facilitating early identification and timely intervention for symptomatic KOA” (aligning with the focus on risk stratification).
2. In the Discussion, it is adjusted to “enhancing the precision of KOA risk stratification” (when addressing predictive models) and “delaying the onset or slowing the progression of symptomatic KOA” (when discussing intervention implications).
   All revisions preserve the original meaning while eliminating redundancy and enriching linguistic variety.

We greatly appreciate your professional guidance, which has significantly improved the clarity, coherence, and conciseness of our manuscript. We hope these revisions address your concerns. We look forward to your further feedback and appreciate your time and consideration.

**Response to Reviewer #1**

**Comment 1:**
*“A limitation of this analysis may be the lack of consideration of physical activity and work performed in the study population.”*

**Response:**
We sincerely thank the reviewer for raising this crucial point. We fully agree that occupational history (e.g., lifelong manual labor) and detailed physical activity levels are important factors that could theoretically influence both biological aging and KOA risk.

Upon careful consideration, we decided not to include these variables in our primary multivariate adjustment models for the following methodological reasons:

1. **Measurement Limitations in CHARLS**: While CHARLS contains data on *current* occupation (e.g., agricultural, non-agricultural, retired), it lacks a detailed, lifetime occupational history that would accurately capture long-term joint loading. Similarly, the dataset does not include objective or finely-grained subjective measures of physical activity (e.g., metabolic equivalent task hours). The available proxies (e.g., basic activities of daily living) are more indicative of health outcomes than of exposure levels and are thus unsuitable as confounders.
2. **Risk of Introduced Bias**: Crucially, including these imperfect proxies could introduce greater bias than it resolves. For instance, current occupation and physical activity levels are not merely confounders; they are also likely *consequences* of health status (**reverse causality**). Individuals with severe knee pain (symptomatic KOA) are much more likely to reduce their physical activity levels or change their occupation. Adjusting for these downstream consequences of the disease would inadvertently adjust away part of the very effect we aim to measure, potentially leading to an underestimation of the true association between biological age and KOA.

Therefore, to maintain the clarity of our analysis and avoid introducing new sources of bias, we focused on adjusting for a robust set of demographic and clinical confounders that are less susceptible to this reverse causality, such as age, sex, BMI, and comorbidities.

We have now explicitly acknowledged this thoughtful point as a limitation in the revised Discussion section *(Please see Page 30, Line 615-621)*

:

*“Fifth, although occupational physical load and activity levels are potential contributors to KOA risk, we did not adjust for them in our primary models. This decision was based on the limitations of the available data (e.g., lack of detailed lifetime occupational history and objective physical activity measures) and the substantial risk of reverse causality, as prevalent KOA severely impacts an individual’s current activity and work capacity. Future prospective studies with dedicated, pre-symptom exposure assessments are needed to disentangle these complex relationships.”*

**Comment 2:**
*“Another interesting aspect is the possible difference between biological and chronological age in the study population, as well as the reference to average life expectancy.”*

**Response:** We thank the reviewer for this insightful suggestion. We have now expanded the discussion to interpret the implications of the discrepancy between BA) and CA in the revised manuscript. A new paragraph has been added to the Discussion section *(Please see Page 26, Line 526-531)* to address the potential link between accelerated biological aging, compressed healthspan, and future risk of disability, which aligns with the reviewer's interest in life expectancy:

*“Furthermore, the divergence betweenBA and CA underscores heterogeneous aging trajectories, likely reflecting cumulative exposure to environmental, lifestyle, and genetic factors. The accelerated risk of symptomatic KOA beyond a BA threshold of ~66.7 years suggests that individuals with advanced biological aging may experience a compressed healthspan, with earlier onset of age-related disorders. Future longitudinal studies are needed to explore whether this BA acceleration predicts overall lifespan reduction or earlier disability.”*

**Response to Reviewer #2**

**Comment 1:**
*The cross-sectional design does not allow causal inference, yet parts of the conclusion* *suggest predictive or causal implications. Recommendation: Reframe claims to emphasize “association” rather than “prediction/causality.”*

**Response:** We are deeply grateful to the reviewer for raising this critical point. We entirely agree that causal inferences are not permitted by our cross-sectional design. In direct response to this comment, we have conducted a **comprehensive and systematic revision** of the entire manuscript to meticulously eliminate any language that could be construed as implying prediction or causality, and to consistently reframe our findings strictly in terms of association.

This revision was not limited to the conclusions but was applied throughout the manuscript, including the Title, Abstract, Introduction, Results, Discussion, and Conclusion sections. Key specific changes include:

1. **Title:** Revised to “Biological Age Threshold **is Associated with** Symptomatic Knee Osteoarthritis Risk...” to set the appropriate tone from the outset.
2. **Abstract:** All predictive claims were replaced. For example, we now state “BA **is strongly and non-linearly associated with** symptomatic KOA risk” and “BA assessment **could inform** future interventional studies” instead of suggesting direct clinical prediction.
3. **Methods:** The machine learning aim was rephrased from “to predict” to “to **assess the association between** features and symptomatic KOA status”.
4. **Results:** Terminology was shifted from “predictive abilities” to “**discriminative abilities**” and from “identifying individuals at risk” to “**distinguishing individuals with**” the condition.
5. **Discussion and Conclusion:** We rigorously scrutinized every statement. Language implying clinical utility (e.g., “predictive value,” “integrate into clinical practice”) was softened to highlight the value for “**risk assessment**” and “**informing future research**.” We have further reinforced the limitation regarding causality in the conclusion.

In essence, we have undertaken a **wholesale transformation** of the manuscript's language to ensure it aligns perfectly with the associative nature of our study design. We hope that these revisions have strengthened the manuscript and thank the reviewer again for your invaluable feedback.

**Comment 2:** *Sample Selection and Representativeness: From ~38,800 participants, only 9,505 remained, and finally 1,000 KOA cases were analyzed. The selection process may introduce bias. Recommendation: Provide detailed explanation on exclusion criteria, missing data handling, and sensitivity analysis.*

**Response:** We sincerely thank the reviewer for raising these critical points regarding sample selection and potential bias. We agree that transparency in this process is essential. We have thoroughly revised the manuscript to address each of your recommendations, as detailed below.

1. Detailed Explanation on Exclusion Criteria:
We have completely rewritten the ‘Study Population’ subsection under ‘Methods’ to provide a stepwise and transparent account of the participant selection process. We now explicitly list the three exclusion criteria applied and, most importantly, specify the number of participants excluded at each step:

- (i) Age <45 years (n = 1,787 excluded)
- (ii) Missing data required to calculate biological age (n = 15,394 excluded)
- (iii) Missing symptomatic KOA status information (n = 12,114 excluded)
  This revision makes it clear that the final analytical cohort of 9,505 participants comprises all individuals with complete data on both the primary exposure (BA) and outcome (symptomatic KOA). We believe this level of detail directly addresses your concern and allows readers to fully appraise the flow of participants. *(Please see Page 8, Line 152-157).*

2. Missing Data Handling:
As requested, we have now added a clear statement in the ‘Study Population’ subsection explaining our approach to handling missing data for the other study variables (covariates). We state: *“For the remaining covariates included in the regression and machine learning models (e.g., lifestyle factors, comorbidities), which had a low rate of missingness (<2%), we employed multiple imputation by chained equations (MICE) to handle missing values and preserve the analytical sample size.”*
The technical details of the MICE implementation were already described in the ‘Machine Learning Models’ section and remain there. This addition ensures that the reader understands our approach to data completeness immediately when reviewing how the cohort was constructed. *(Please see Page 8, Line 158-169).*

3. Sensitivity Analysis:
We thank the reviewer for suggesting a sensitivity analysis. We have carefully considered this point and concluded that conducting a meaningful sensitivity analysis of the type typically used to assess selection bias (e.g., comparing the association between BA and symptomatic KOA among included versus excluded participants) is not directly feasible in this context.

The primary reason is that the exclusion criteria were based precisely on the absence of the core variables required for our analysis—biological age and symptomatic KOA status. Since these variables are unavailable for the excluded groups, we are unable to evaluate whether the relationship between BA and KOA differs between those included and those excluded.

In place of a formal sensitivity analysis, we have taken steps to enhance transparency and acknowledge this limitation explicitly. We have included a detailed description of the exclusion process and added the following statement in the Limitations section of the Discussion:

*“Fourth, the multi-stage participant selection process, necessary to obtain a sample with complete data for the core variables (biological age and KOA status), may have introduced selection bias. A direct sensitivity analysis comparing the exposure-outcome relationship between included and excluded participants was not possible due to the lack of data on these core variables in the excluded groups. Although the final analytical sample retains key characteristics of the original CHARLS cohort, this potential bias may affect the representativeness of our sample and limit the generalizability of our findings.”* *(Please see Page 30, Line 610-615).*

We hope that this candid explanation, along with the additional methodological details provided, adequately addresses the reviewer’s concern regarding potential selection bias.

Once again, we are grateful for these insightful comments, which have significantly improved the clarity and rigor of our manuscript.

**Comment 3:** *Symptomatic KOA was defined solely by self-reported physician diagnosis with pain, lacking imaging confirmation (e.g., KL grading). Recommendation: Discuss the risk of misclassification in greater detail and, if possible, validate findings against other diagnostic definitions.*

**Response:** We sincerely thank the reviewer for this critical comment. We fully acknowledge that our definition of *symptomatic KOA*—based on *self-reported physician diagnosis* accompanied by knee pain and lacking radiographic confirmation (e.g., *Kellgren-Lawrence grading*)—is a limitation that may introduce *misclassification bias*.

As recommended, we have now discussed this risk in greater detail in both the **Methods** (under “Variable Definitions”) *(Please see Page 9, Line 182-184)* and **Discussion** sections *(Please see Page 29, Line 596-601)*. We explicitly state that this widely used epidemiological definition, while feasible for large-scale studies, may lead to non-differential misclassification, potentially attenuating the observed effect sizes toward the null.

Regarding the valuable suggestion to *validate findings against other diagnostic definitions*, we regret that this is not feasible with the current CHARLS dataset, as it does not contain imaging or alternative clinical exam data for the same participants. Therefore, we could not perform analyses using stricter radiographic criteria.

We have strengthened the manuscript by acknowledging this limitation more explicitly and believe that, despite it, our findings provide valuable insights into the *association* between *BA* and clinically meaningful *symptomatic KOA* at the population level.

Thank you again for this constructive feedback.

**Comment 4:** *Only 8 biomarkers were used instead of the standard 12 in KDM-BA. Although justified with prior validation, potential bias is not sufficiently addressed. Recommendation: Provide comparative or sensitivity analysis, and acknowledge limitations more explicitly.*
**Response:** We thank the reviewer for raising this important methodological point. We agree that the use of a reduced set of biomarkers is a limitation that warrants careful consideration, and we have revised the manuscript to address this concern more explicitly.

1. Justification Based on Prior Validation:
The reviewer correctly notes that the standard KDM algorithm incorporates 12 biomarkers. The CHARLS database, however, does not include all of them (specifically, albumin, red blood cell count, ferritin, and transferrin are unavailable). Our selection of the remaining 8 biomarkers was not arbitrary but was guided by established validation studies conducted in the Chinese population. As cited in our manuscript, the works of Liu (2021) [Ref 18] and Ruan et al. (2025) [Ref 19] specifically demonstrate that this subset of biomarkers reliably captures the construct of biological aging and maintains predictive validity for health outcomes in this demographic context. Therefore, we employed a method that, while a subset, has been shown to be valid for the population under study.

2. Infeasibility of Comparative Analysis and Focused Revision:
We sincerely appreciate the reviewer's suggestion for a comparative or sensitivity analysis. We must note, however, that a direct head-to-head comparison between the 8- and 12-biomarker algorithms is not feasible within the CHARLS dataset because the four additional required biomarkers were not measured, presenting an inherent constraint of secondary data analysis.

In lieu of an analysis that cannot be performed, we have focused on fully addressing the spirit of the reviewer's comment by explicitly acknowledging this limitation and its potential implications. We have substantially revised the Discussion section to incorporate a more direct and detailed statement regarding this issue. The added text reads: *(Please see Page 30, Line 602-607)*.

*“Third, the BA calculation relied on a reduced set of 8 systemic biomarkers instead of the standard 12 used in the KDM method, due to data availability constraints in CHARLS. Although prior studies have validated similar reduced sets in Chinese populations [18,19], this approach may introduce bias and affect the absolute value of BA estimates, potentially limiting direct comparability with studies using the full biomarker panel. Future research should aim to utilize the complete set of biomarkers where possible.”*

We are grateful for this suggestion, which has improved the transparency of our manuscript.

**Comment 5:**
*“The sample size (1,000 KOA cases) may be limited for complex models, raising concerns of overfitting. No external validation dataset was used. Recommendation: Add stronger justification for model selection, expand on overfitting prevention, and clarify whether performance was robust across subsets.”*

**Response:** We sincerely thank the reviewer for raising these important methodological concerns. We have now strengthened the manuscript to address each point as follows:

1. **Sample Size and Overfitting Prevention:**
   Although the sample size of symptomatic OA cases is modest (n=1,000), we implemented multiple robust strategies to mitigate overfitting:
   - A 70/30 train-test split was used to evaluate model performance on unseen data.
   - Five-fold cross-validation was employed for hyperparameter tuning and to ensure stability.
   - Regularization techniques (L1/L2) and early stopping were integrated into gradient boosting models (XGBoost, LightGBM, CatBoost).
   - Feature selection via LASSO regression was applied to reduce dimensionality and enhance model interpretability.

These measures are now explicitly described in the Methods section (subsection “Machine Learning Models”). The consistent AUROC values (>0.90) across cross-validation folds (Table 4, Figure 6) further support the robustness of our models.

2. **External Validation:**
   We acknowledge that no external validation dataset was available. To compensate, we performed rigorous internal validation through cross-validation and subgroup analyses, which showed consistent model performance. We have added a statement in the Discussion regarding this limitation and the need for external validation in future studies.
3. **Model Justification and Subgroup Robustness:**
   We selected tree-based ensemble models (e.g., XGBoost) for their ability to handle non-linear relationships and interaction effects, which are common in biological aging and symptomatic OA risk. The models performed consistently well across cross-validation folds and subgroups, indicating generalizability within the cohort.

Thank you again for this constructive feedback.

The following paragraph has been added to the Methods section *(Please see Page 12, Line 243-256)* under “Machine Learning Models” to detail the overfitting prevention strategies:

*“Feature selection was performed using LASSO regression, which identified non-zero coefficient predictors for model inclusion. Prior to modeling, continuous variables (e.g., BA, BMI, blood biomarkers) were standardized using z-scores (mean = 0, SD = 1), and categorical variables (e.g., sex, education) were one-hot encoded. Covariates with minimal missingness (<2%) were handled via multiple imputation by chained equations (MICE). To mitigate overfitting given the sample size, the dataset was split into a 70% training set and a 30% testing set stratified by KOA status. Model implementation details were as follows: Decision Tree and Random Forest used the Gini impurity criterion; gradient boosting models (XGBoost, LightGBM, CatBoost) were tuned for maximum depth, learning rate, and number of estimators, incorporating L1/L2 regularization and early stopping (50 rounds); and Support Vector Machine (SVM) employed a radial basis function (RBF) kernel with regularization parameter C tuned via five-fold cross-validation. These measures contributed to stable model performance, as evidenced by consistent AUROC values (>0.90) across cross-validation folds.”*

Additionally, the following statement has been added to the Discussion section *(Please see Page 31, Line 621-626)* within the Limitations subsection:

*“Furthermore, although we performed rigorous internal validation through cross-validation and subgroup analyses, the lack of an independent external dataset for validation means that the generalizability of the observed associations may be limited. Future studies in larger, multi-center cohorts and diverse populations are needed to replicate these associations and confirm the robustness of BA as a marker for symptomatic KOA risk assessment.”*

**Comment 6:**
*Discussion and Interpretation: The discussion overstates the clinical applicability of BA without sufficient consideration of cost, feasibility, and generalizability. Recommendation: Temper the claims and include comparison with international studies.*

**Response:** We are grateful to the reviewer for this crucial feedback. We agree that the *clinical applicability* of *biological age* requires careful consideration of practical challenges.

In response to this comment, we have substantially revised the **Discussion** section to:

1. **Temper our claims** regarding immediate clinical translation. We now explicitly acknowledge the challenges related to *cost*, *feasibility* (e.g., measuring multiple biomarkers), the need for *population-specific validation* of BA algorithms, and limited *generalizability* to other healthcare settings. *(Please see Page 29, Line 579-582)*
2. **Include a direct comparison with international studies**. We have integrated findings from two recent cross-sectional studies utilizing the U.S. *NHANES* database, which also employed KDM-based biological aging measures and reported consistent positive associations with osteoarthritis risk. This addition strengthens our discussion by situating our findings within the broader international context and supports the robustness of the observed association. *(Please see Page 25, Line 512-522)*

We believe these revisions have resulted in a more balanced and nuanced discussion that accurately reflects the current *potential* of BA for risk stratification while honestly addressing the practical hurdles that must be overcome before its widespread clinical implementation.

Thank you for this insightful suggestion, which has significantly improved our manuscript.

**Comment 7:**
*Minor Concerns*

*Some terms in the abstract and conclusion (e.g., “predicts,” “causal”) should be replaced with more cautious wording.*

*Clarify covariates included in each logistic regression model table to improve transparency.*

*Figures (e.g., RCS, SHAP plots) should include clearer axis labels and clinical interpretation.*

*Data availability statement should specify exact CHARLS dataset versions used.*

**Response:** We extend our sincere thanks to the reviewer for these exceptionally constructive and detailed suggestions. We have meticulously addressed each point, and the revisions have enhanced the clarity, rigor, and overall quality of our manuscript. Below is a point-by-point summary of our responses and the actions taken.

**1. & 2. Regarding cautious wording and covariate clarification:**
We thank the reviewer for underscoring the importance of precise language and full transparency. In direct response:

- We have performed a **systematic, full-manuscript review** to eliminate any language that could imply causation or premature prediction, strictly reframing our findings in terms of **association** and **discriminative ability**. This involved revisions to the Title, Abstract, Methods, Results, Discussion, and Conclusion.
  - **Specifically in the Abstract,** we replaced the verb “predicted” with the more accurate phrase “**were used to distinguish cases of**” symptomatic KOA. All conclusive language now emphasizes robust association.
- To maximize transparency in our regression analyses, we have now **explicitly listed all covariates** included in each adjustment model directly in the footnote of **Table 2**: *(Please see Page 17, Line 318-323)*
  - **Model 1: Adjusted for none.**
  - **Model 2: Adjusted for gender, education, and residence.**
  - **Model 3: Adjusted for Model 2 combined with BMI, cancer, smoking, drinking, hypertension, dyslipidemia, and CVD.**

This clarification ensures readers can fully appraise the rigor of our analytical approach.

**3. Regarding figure clarity and clinical interpretation:**
We greatly appreciate this suggestion, which has substantially improved the accessibility of our key findings.

- **Figure 3 (RCS plot):** We have regenerated this figure with **clearer axis labels and font sizes** to improve readability. Furthermore, we have **completely rewritten its caption** to provide a detailed statistical and clinical interpretation. It now explicitly states the location of the knots, the reference point, and, crucially, the **clinical implication of the identified BA threshold (~66.7 years)** for risk escalation.
- **SHAP Plots (Figures 7, 8, 9):** We have revised all relevant captions and the accompanying text in the “**Feature Contributions in the XGBoost Model**” section *(Please see Page 22, Line 423-446).* The descriptions now focus on how each feature **contributes to the model’s output** and its **association with the outcome**.

**4. Regarding the Data Availability statement:**
We thank the reviewer for highlighting this oversight. We have now updated the “**Data Availability**” section to specify the exact dataset versions used:
“The data were obtained from the China Health and Retirement Longitudinal Study (CHARLS) 2011 baseline survey and 2015 follow-up survey datasets (**Version: CHARLS_2011_v1.5 & CHARLS_2015_v1.0**), which are publicly available at <https://charls.pku.edu.cn/index.htm>.” *(Please see Page 33, Line 669-672).*

We have carefully considered all comments and revised the manuscript accordingly. We hope our responses and the changes made have adequately addressed the points raised. Thank you once again for the valuable insights, which have undoubtedly strengthened our work.

**Reviewer #3:**

**Comment 1:**
*Strengthen international comparison in the literature review. The introduction is mostly focused on the Chinese population and CHARLS data. While this is aligned with the study's aims, it limits international contextualization. I suggest including a brief overview of international research on the relationship between biological age and KOA—especially regarding differences in BA construction methods and clinical applications—to enhance the theoretical richness and global relevance of the study.*

**Response:** We thank the reviewer for this excellent suggestion, which has helped us improve the global context of our manuscript. We agree that providing an international perspective enriches the theoretical foundation of our work.

In direct response to this comment, we have revised the **Introduction** to include a concise overview of international research on *biological age*. Specifically, we have added a new section in the second paragraph that: *(Please see Page 5 Line 88-93)*

1. Explicitly mentions *global* methods for estimating BA (e.g., *KDM*, *PhenoAge*), acknowledging the differences in biomarker panels and construction methodologies.
2. Cites international evidence (references [8-11]) demonstrating that the association between *accelerated BA* and age-related diseases (including *osteoarthritis*) is a consistent finding across different populations.

We believe this addition successfully strengthens the *international contextualization* of our study, enhancing its *theoretical richness* and *global relevance* by framing our research within the broader international discourse on biological aging, while maintaining a clear focus on our primary aim within the Chinese population.

Thank you for this valuable recommendation.

**Comment 2:**

*Clarify model structure and data preprocessing in the machine learning section: Although the manuscript evaluates six machine learning models, there is limited detail regarding model construction. Please clarify how features were selected, whether feature scaling or normalization was applied, how missing data were handled, and how overfitting was prevented. These additions would enhance reproducibility and model transparency.*

**Response:** We sincerely thank the reviewer for this critical comment. We agree that greater methodological detail is essential for reproducibility. In direct response, we have comprehensively revised the **'Machine Learning Models'** subsection in the Methods to include all the requested details: *(Please see Page 12, Line 243-256).*

- **Feature Selection:** The specific predictors were identified through **LASSO regression**, which selected non-zero coefficient features for model inclusion.
- **Feature Scaling & Missing Data:** We have clarified that all continuous variables (e.g., BA, BMI) were **standardized using z-scores**, categorical variables were **one-hot encoded**, and covariates with minimal missingness (<2%) were handled using **multiple imputation by chained equations (MICE)**.
- **Model Construction:** We now specify key implementation details for each algorithm: **Decision Tree/Random Forest** used the Gini impurity criterion; **Gradient Boosting models** (XGBoost, LightGBM, CatBoost) were tuned for depth, learning rate, and number of estimators with **L1/L2 regularization and early stopping**; and **SVM** employed an **RBF kernel** with cross-validated regularization.
- **Overfitting Prevention:** The paragraph now explicitly integrates our strategies, including the **70/30 train-test split** and **five-fold cross-validation**.

We hope these revisions have improved the transparency and reproducibility of our machine learning workflow by explicitly addressing the points you raised. Thank you for helping us improve the manuscript.

**Comment 3:**
*Add a brief discussion of potential clinical applications of BA. While the predictive value of BA is emphasized, the practical implications are underdeveloped. I recommend briefly discussing how BA could potentially be integrated into clinical KOA screening or aging-related health assessments, such as early warning systems or personalized intervention strategies, to enhance the manuscript’s translational value.*

**Response:** We sincerely thank the reviewer for this excellent suggestion, which has significantly enhanced the translational impact of our manuscript.

As recommended, we have now added a **brief discussion on the potential clinical applications** of *biological age* in the latter part of the **Discussion** section *(Please see Page 29, Line 582-589)* and the **Conclusion**. *(Please see Page 32, Line 645-652)* We specifically discuss how BA assessment *could be integrated* into clinical practice, such as *primary care* or *geriatric health screenings*, to serve as an *early warning* tool for identifying high-risk individuals. This would facilitate the initiation of *personalized intervention strategies* (e.g., *lifestyle modifications* or enhanced monitoring) well before the onset of severe symptoms.

We believe this addition successfully addresses the reviewer's concern by moving beyond purely predictive value to articulate the potential practical utility of BA in *clinical KOA screening* and *aging-related health assessments*, thereby strengthening the manuscript's *translational value*.

Thank you for this constructive comment.

**Comment 4:**
*Unify terminology and maintain consistency throughout the manuscript. The manuscript inconsistently uses terms like “biological age,” “BA,” and “KDM-BA.” I suggest defining the term clearly at first mention and consistently using “BA (KDM-based)” or another unified format throughout the paper to improve clarity and professionalism.*

**Response:** We sincerely thank the reviewer for this meticulous comment, which has significantly improved the clarity and professionalism of our manuscript. We completely agree that consistent terminology is crucial.

In direct response to this feedback, we have now thoroughly revised the entire manuscript to ensure **strict terminological consistency**:

1. We have clearly defined the term at its first mention in both the *Abstract* and *Introduction* as *“biological age (BA, calculated using the Klemera-Doubal method [KDM])”*.
2. Throughout the rest of the manuscript, including the *Methods*, *Results*, *Discussion*, *Figures*, and *Tables*, we now **consistently use the abbreviation “BA”** after its initial definition. We have replaced nearly all instances of the full term “biological age” and any variant notations (e.g., *KDM-BA*) with the unified abbreviation “BA” for enhanced readability and flow.

We believe these revisions have successfully resolved the inconsistency and greatly improved the manuscript's clarity. We are grateful for this valuable suggestion.

**Comment 5:**
*Provide clearer explanations for SHAP interpretation plots. Although SHAP plots are visually compelling, the textual explanations are brief. I suggest elaborating on what the colors (high/low feature values), directions (positive/negative effects), and spread (magnitude of influence) mean in the context of SHAP bar and beeswarm plots. This would help readers better interpret model results.*

**Response:** We thank the reviewer for this excellent suggestion, which has greatly improved the interpretability of our machine learning results. We agree that a more detailed explanation of the *SHAP plots* is essential for readers to fully understand the model's decision-making process.

In direct response to this comment, we have expanded the textual explanations in the “**Feature Contributions in the XGBoost Model**” subsection of the **Results**. We now provide a detailed elaboration on: *(Please see Page 22, Line 424-446)*

- **Colors**: We explicitly state that in the beeswarm plot, *color intensity* reflects *feature values* (“*with purple indicating high values and yellow low values*”).
- **Directions**: We clearly define the meaning of the *horizontal axis* (“*positive values increase the predicted risk of KOA, whereas negative values decrease it*”).
- **Spread**: We interpret the *horizontal spread* of points as reflecting the “*variation in influence across individuals*”, and use the dispersion of *BA* points to illustrate its “*consistently positive yet heterogeneous effect*”.

We believe these additions provide the necessary context for readers to independently and accurately interpret the *SHAP bar and beeswarm plots*, thereby enhancing the clarity and value of our model interpretation. Thank you for this constructive feedback.

**Comment 6:**

*To increase transparency and interpretability, I suggest including histograms or boxplots of key input variables (e.g., CRP, SBP, HbA1c, etc.) and BA itself in the supplementary materials. This would allow readers to assess data skewness, outliers, and distribution assumptions.*
**Response:** We thank the reviewer for this excellent suggestion. We agree that this will enhance the transparency of our work.

*As requested, we have now included histograms for all eight key input variables—CRP, SBP, HbA1c, total cholesterol, triglycerides, urea nitrogen, creatinine, and platelet count—as well as for the calculated Biological Age (BA) itself as* ***Supplementary Figures S1 to S9****.* *(Please see Page 10, Line 197-200)*

These new supplementary figures allow readers to visually assess the distribution of each variable used in our analysis. We believe this addition significantly improves the interpretability of our study. Thank you again for this constructive feedback.

**Comment7:**

*Streamline the machine learning result presentation. The current machine learning results section is somewhat overloaded with detailed fold-level performance metrics. I suggest moving some of the five-fold cross-validation details (e.g., individual fold metrics) to supplementary materials, while retaining only the averaged summary metrics (e.g., AUROC, accuracy, F1-score) in the main text for better readability and flow.*

**Response:** We thank the reviewer for this valuable suggestion. We have now revised the manuscript to streamline the presentation.

As recommended, we have moved the detailed fold-level performance metrics from Table 4 to the Supplementary Materials *(now Supplementary Table S1)*. In the main text, *we have replaced the original table with a concise version that retains only the mean AUROC, accuracy, and F1-score* from the 5-fold cross-validation. This change significantly improves the readability of the section while preserving all necessary information for the interested reader.

We believe the revised manuscript now presents the machine learning results in a more clear and focused manner.

We sincerely thank the reviewer for the insightful and constructive comments. These suggestions have been instrumental in strengthening the international perspective, methodological rigor, and overall clarity of our manuscript. We are grateful for the valuable contributions, which have greatly improved the quality of our work.
